# 大语言模型辅助文献计量分析解读的可行性与局限性探索研究——以血友病基因治疗研究热点挖掘为例

**DOI:** 10.3760/cma.j.cn121090-20260122-00046

**Published:** 2026-07

**Authors:** 杨 白, 鸿文 薄, 峰 薛, 迎 王, 靖 张, 振 宋

**Affiliations:** 1 中国医学科学院血液病医院（中国医学科学院血液学研究所），血液与健康全国重点实验室，国家血液系统疾病临床医学研究中心，细胞生态海河实验室，天津 300020 State Key Laboratory of Experimental Hematology, National Clinical Research Center for Blood Diseases, Haihe Laboratory of Cell Ecosystem, Institute of Hematology & Blood Diseases Hospital, Chinese Academy of Medical Sciences & Peking Union Medical College, Tianjin 300020, China; 2 天津医学健康研究院，天津 301600 Tianjin Institutes of Health Science, Tianjin 301600, China

**Keywords:** VOSviewer, CiteSpace, Deepseek, 血友病, 基因治疗, VOSviewer, CiteSpace, DeepSeek, Hemophilia, Gene therapy

## Abstract

**目的:**

评价DeepSeek辅助解读文献计量分析结果的可行性及局限性。

**方法:**

检索Web of Science核心合集数据库1970–2024年发表的血友病基因治疗相关英文论文，共纳入1 599篇，其中美国、中国和英国文献分别为1 019、174和172篇。采用VOSviewer对全球及美国、中国和英国文献进行关键词共现与聚类分析，采用CiteSpace对全球文献进行关键词共现、聚类及突现分析。将软件导出的结构化数据输入DeepSeek-R1-0528，由其概括研究热点及演进趋势。邀请2名领域专家基于相同数据独立解读全球研究热点及演进趋势，并审查DeepSeek对3个国家研究热点的解读结果。针对CRISPR/Cas9、干细胞和宫内基因治疗等方向开展补充文献检索，对模型提出的国家研究特色进行交叉验证。

**结果:**

DeepSeek将全球血友病基因治疗研究概括为4个主要方向：基因治疗载体的设计与体内表达调控、腺相关病毒载体递送策略及免疫原性突破、基因治疗临床转化与真实世界应用、免疫耐受诱导及抑制剂形成机制。依据CiteSpace生成的7个主要聚类及关键词时间和突现信息，DeepSeek进一步将该领域的发展概括为早期载体及动物模型探索、递送与治疗策略优化、临床验证以及临床应用与患者获益关注4个阶段。对于全球文献数据，DeepSeek对核心研究主题和总体发展脉络的判断与领域专家基本一致，且能够较快形成结构清晰的概括结果。但其在专业术语、阶段划分及历史里程碑识别方面仍需专家校准。对于单一国家文献数据，DeepSeek存在概念混淆，对新兴方向过度概括，以及依据国家内部关键词频次或聚类强度进行不当跨国比较的风险。补充检索表明，单一国家数据集中的关键词相对突出或缺失，不能直接解释为该国在相应方向上领先或缺乏研究。

**结论:**

大语言模型可辅助解读全球层面的文献计量分析结果，提高研究热点识别和发展脉络梳理的效率，但不能替代领域专家。对于新兴研究方向和跨国比较，应结合专业审核，并引入统一检索口径下的全球参照数据进行校准和交叉验证。

## Abstract (en)

**Objective:**

To evaluate the feasibility and limitations of using a large language model to assist in the interpretation of bibliometric findings.

**Methods:**

This study retrieved 1599 English-language articles on hemophilia gene therapy published between 1970 and 2024 from the Web of Science Core Collection, comprising 1019 articles from the United States, 174 from China, and 172 from the United Kingdom. VOSviewer was used for keyword co-occurrence and clustering analyses of the global and country-specific datasets, whereas CiteSpace was used for keyword co-occurrence, clustering, and burst-detection analyses of the global dataset. Structured data exported from the two tools were interpreted with DeepSeek-R1-0528 to summarize research hotspots and their temporal evolution. Two domain experts independently interpreted the global data and reviewed the country-specific interpretations generated using DeepSeek. Additional literature searches on CRISPR/Cas9, stem-cell-based approaches, and in utero gene therapy were conducted to cross-validate the national research characteristics inferred by the model.

**Results:**

DeepSeek summarized global hemophilia gene therapy research into four major themes: the design and in vivo expression regulation of gene therapy vectors; Adeno-associated virus vector delivery strategies and breakthroughs in immunogenicity; the clinical translation and real-world application of gene therapy; the mechanisms of immune tolerance induction and inhibitor formation. Based on seven major CiteSpace clusters and the temporal and burst information of the keywords, DeepSeek further outlined four stages of development: early exploration of vectors and animal models, optimization of delivery and therapeutic strategies, clinical validation, and increasing attention to clinical application and patient benefits. The core research themes and overall developmental trajectory for the global dataset identified using DeepSeek were broadly consistent with expert interpretations, and the model rapidly generated well-structured summaries. However, expert calibration remained necessary for professional terminology, stage delineation, and historical milestone recognition. In country-specific datasets, DeepSeek demonstrated risks of conceptual conflation, overgeneralization of emerging research directions, and inappropriate cross-country comparisons according to within-country keyword frequencies or cluster strengths. Additional searches revealed that the relative prominence or absence of a keyword within a national dataset could not be directly interpreted as international leadership or a lack of research activity.

**Conclusion:**

Large language models can assist in interpreting global bibliometric findings and improve the efficiency of identifying research hotspots and outlining developmental trajectories; however, they cannot replace domain experts. Interpretations of emerging research directions and cross-country differences require expert review and calibration using globally comparable data obtained under a unified search strategy.

随着血友病基因治疗相关基础研究和临床试验不断推进，该领域的文献数量持续增加，研究主题也逐渐由载体构建和基因表达向治疗效果、安全性、免疫反应及长期随访等多个方向拓展[1]–[4]。如何从大量文献中系统识别研究热点、梳理主题之间的关联及其演变脉络，对于研究人员把握学科进展和确定后续研究方向具有重要意义。

关键词共现分析是文献计量学中识别研究热点和主题结构的常用方法。借助VOSviewer、CiteSpace等工具，可以构建关键词共现网络，并根据关键词之间的关联形成主题聚类。然而，软件生成的聚类标签、高频关键词及网络关系并不能直接等同于具有明确领域含义的研究主题。对聚类内容进行准确命名和解释，通常还需要结合代表性文献，并依赖文献计量学人员与领域专家的共同判断，因而存在分析工作量较大、解释过程主观性较强等问题。

人工智能（artificial intelligence，AI），特别是大语言模型，在文本归纳、语义关联和多文献内容整合等方面表现出一定潜力，为辅助解读文献计量分析结果提供了新的技术路径。AI可以基于聚类关键词及相关文献，对聚类主题进行概括，并辅助梳理不同研究主题之间的联系及其时间变化。但AI生成内容仍可能受到输入信息、提示方式和模型自身局限的影响，其在文献计量结果解读中的应用方式及可靠性仍有必要进一步探索。

本研究收集Web of Science（WOS）核心合集数据库收录的血友病基因治疗相关文献，采用VOSviewer和CiteSpace开展关键词共现、聚类及突现分析，并利用大语言模型（本研究采用DeepSeek）对全球及主要国家的研究热点和全球研究热点的演变过程进行辅助解读。在此基础上，通过领域专家对DeepSeek解读结果进行验证和审查，分析其在文献计量结果解读中的应用价值及可能存在的局限，为血友病基因治疗领域研究热点的识别和发展脉络的梳理提供文献层面的参考。

## 资料与方法

一、数据采集

检索WOS核心合集数据库采集血友病基因治疗研究领域的论文，检索式为TS＝（"gene therap*" AND hemophilia），出版年限定为1970–2024年，文献类型限定为“Article”，语言限定为英语，共检索获得1 599篇文献。其中，美国文献1 019篇、中国文献174篇、英国文献172篇。

二、文献计量工具进行关键词共现分析

采用VOSviewer和CiteSpace两种常用文献计量分析工具进行关键词共现分析。关键词分析类型选择全部关键词。VOSviewer分析时，采用同样的词表进行数据清洗，共现网络图选取词频为权重参数。CiteSpace分析时，每一年度进行时间切片，关键词共现分析后，进行关键词突现性检测。

三、DeepSeek进行研究热点及演化趋势解读

1. 输入数据及格式：将VOSviewer和CiteSpace导出的关键词分析结果整理为xlsx格式文档，作为DeepSeek的输入数据，具体数据见附录文件1（**扫描本文首页二维码查阅**）。附录文件共包括5个工作表，分别为CiteSpace全球关键词分析数据，以及VOSviewer全球、美国、中国和英国关键词分析数据。本研究于2025年10月使用DeepSeek-R1-0528模型进行分析。

在利用DeepSeek解读VOSviewer分析结果时，分别从全球、美国、中国和英国文献的map文件中提取Label、Cluster和Weight<Occurrences>3个字段。其中，Label表示关键词，Cluster表示关键词所属的聚类，Weight<Occurrences>表示关键词在相应文献集中出现的次数。各数据表按照聚类编号排列。全球文献数据集包含518个关键词，美国、中国和英国文献数据集分别包含357、75和70个关键词。

在利用DeepSeek解读CiteSpace分析结果时，从关键词共现、聚类及突现分析结果中提取Freq、Label、Year、Burst、BurstBegin、BurstEnd和ClusterID字段。其中，Freq表示关键词出现频次，Label表示关键词，Year表示关键词首次出现年份，Burst表示关键词的突现强度，BurstBegin和BurstEnd分别表示突现的起始年份和结束年份，ClusterID表示关键词所属的聚类。共整理获得714条关键词记录，其中119个关键词的突现强度大于0；Burst值为0表示该关键词未被识别为突现关键词，其BurstBegin和BurstEnd字段为空。根据聚类规模，选取#0～#6共7个主要聚类进行研究热点演化分析。

2. 提示词：

（1）“我检索了Web of Science收录的到2024年底的［全球/美国/中国/英国］血友病基因治疗文献，这里用VOSviewer做了关键词共现分析，共分出了［4/3/3/2］个Cluster，现在表格中有Label、Cluster和Occurrences三列，其中Label列代表关键词，Clutser列代表关键词所属的聚类，Occurrences代表关键词在这段时间在相应文献数据集中出现的次数，此表按照Cluster升序排列，请以血友病临床诊疗专家和基因编辑专家的双重身份，以这个聚类下的关键词信息和关键词共现的次数为依据，以标题总结每个聚类的研究热点”。并将VOSviewer的map数据上传给DeepSeek。

（2）“我是一个文献计量专家，检索了Web of Science核心合集中收录的最早一直到2024年度的血友病基因治疗的研究文献，通过CiteSpace的文献关键词共现分析，关键词突显分析，希望获得领域内研究热点的发展脉络，现在提供的CiteSpace导出的文献，包含Freq参数，代表关键词在检索论文中出现的词频，Label参数，代表关键词名称，Year参数，代表关键词首次在检索到的研究论文中出现的时间，ClusterID参数，代表关键词所属的关键词共现聚类，Burst参数，代表关键词突现程度，数值越大，代表突然受到研究者关注的程度越高，并且BurstBegin和BurstEnd分别代表关键词突然受到关注的起始时间和结束时间。我希望你现在作为一个基因治疗血友病领域专家，对我提供的文献计量数据进行专业领域的解读，解读必须以我提供的原始数据为依据，帮我梳理此领域的研究热点及发展趋势”。并将CiteSpace的关键词共现的网络图数据上传给DeepSeek。

四、领域专家对DeepSeek解读研究热点与演化趋势的验证

邀请2名从事血友病基因治疗相关研究的领域专家，对DeepSeek解读结果进行专业评价。

1. 对全球研究热点及其演化趋势的评价：将VOSviewer导出的全球血友病基因治疗关键词、聚类及出现次数数据，以及CiteSpace导出的全球关键词频次、聚类、首次出现年份和突现信息，分别提供给2名领域专家。专家在未查看DeepSeek解读结果的情况下，依据相同的文献计量数据独立概括主要研究热点及其演化趋势。随后，将专家解读结果与DeepSeek解读结果进行比较，评价二者在研究主题识别、阶段划分及发展趋势判断等方面的一致性和差异。

2. 对美国、中国和英国研究热点解读结果的评价：将DeepSeek对美国、中国和英国血友病基因治疗研究热点的解读结果及相应的VOSviewer关键词聚类数据提供给2名领域专家，由专家从聚类主题概括的准确性、专业术语使用、关键词与主题的对应关系，以及是否存在概念混淆、过度概括或超出原始数据推断等方面进行审查，并记录专家提出的修改意见。

## 结果

一、血友病基因治疗研究文献分布情况

对全球1 599篇血友病基因治疗相关论文的发表时间分布进行分析发现，该领域相关文献最早可追溯至1987年，2000年前后发文量开始稳步增加，2016年后进入较快增长阶段（图1A）。

**图1:**
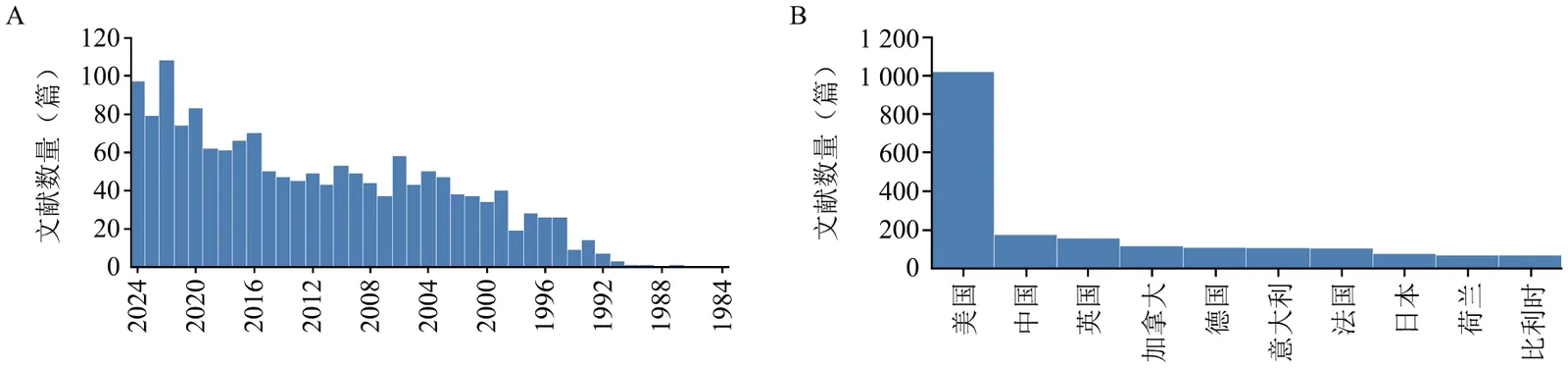
全球血友病基因治疗相关文献的发表情况 **A** 全球血友病基因治疗相关文献发文时间趋势图；**B** 全球血友病基因治疗相关文献发文量排名前10位的国家

从国家分布来看，图1B展示了血友病基因治疗研究发文量排名前10位的国家。美国以1 019篇位居首位，占全球发文量的63.7％；其次为中国（174篇）和英国（172篇）。美国发文量约为中国、英国的6倍。鉴于美国、中国和英国在该领域发文量位居前列，后续分别对3国文献的关键词进行了共现分析，以比较其研究热点特征。

二、VOSviewer关键词共现分析的DeepSeek解读

通过VOSviewer对全球1 599篇血友病基因治疗相关文献进行关键词共现分析，共形成4个聚类（图2A）。DeepSeek依据各聚类所包含的关键词及其出现次数，将4个聚类分别概括为“基因治疗载体的设计与体内表达调控”“AAV载体递送策略及免疫原性突破”“基因治疗临床转化与真实世界应用”“免疫耐受诱导及抑制剂形成机制”（表1）。

**图2:**
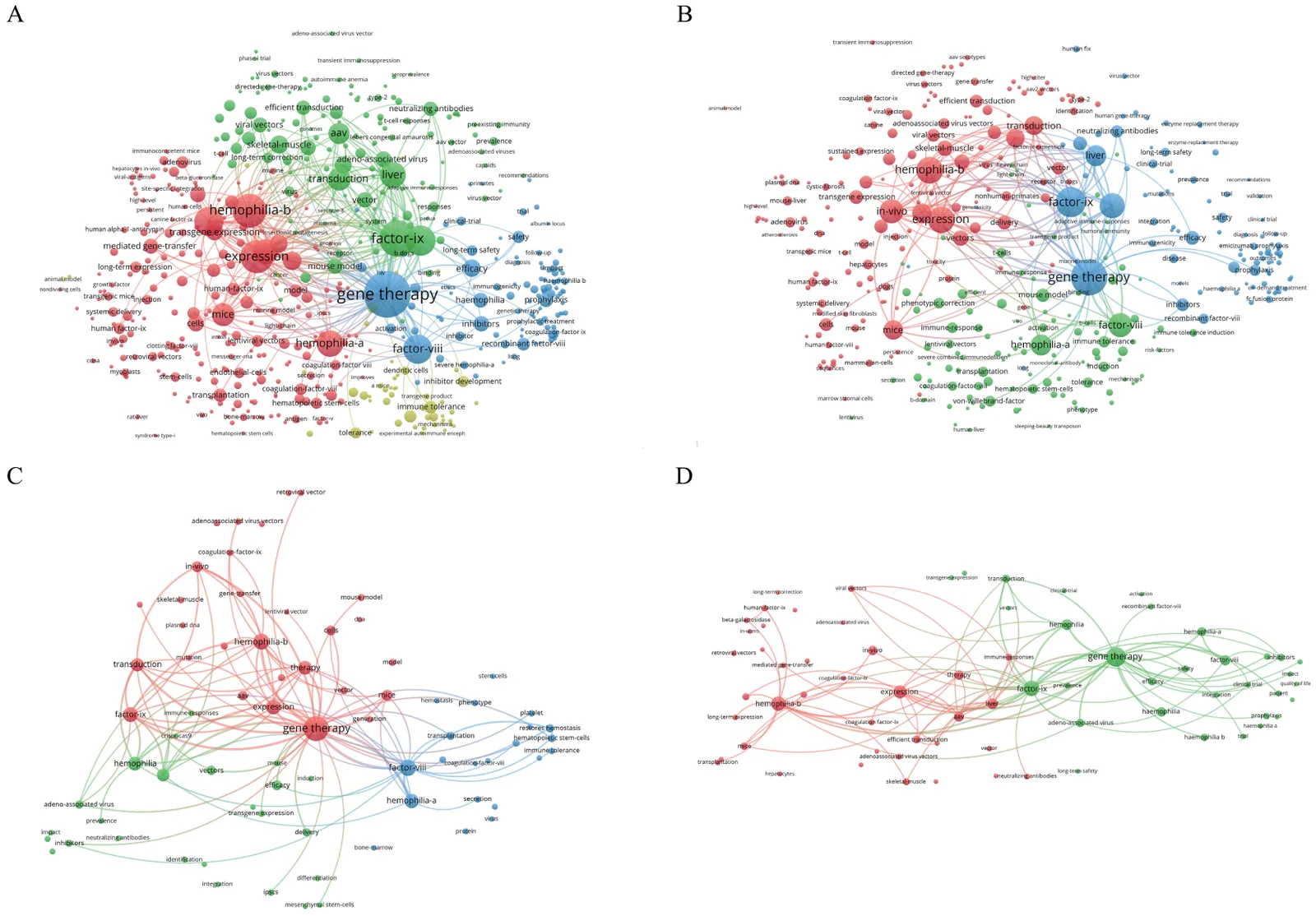
血友病基因治疗关键词共现网络图（VOSviewer） **A** 全球1 599篇文献数据，共四个聚类；**B** 美国1 019篇文献数据，共三个聚类；**C** 中国174篇文献数据，共三个聚类；**D** 英国172篇文献数据，共两个聚类

**表1:** 基于VOSviewer关键词聚类数据的DeepSeek研究热点解读结果

研究热点聚类	全球 （1 599篇）	美国 （1 019篇）	中国 （174篇）	英国 （172篇）
聚类1	基因治疗载体的设计与体内表达调控	B型血友病基因递送系统的优化与转化研究	AAV载体介导的FⅨ基因治疗体系构建与表达调控	AAV载体技术与胎儿期治疗探索
聚类2	AAV载体递送策略及免疫原性突破	FⅧ抑制物清除策略及细胞治疗新路径	免疫屏障突破与细胞疗法新路径	基因治疗临床转化与真实世界管理
聚类3	基因治疗临床转化与真实世界应用	基因治疗临床应用安全监测与卫生经济学评价	FⅧ分泌体系重构及干细胞移植治疗	
聚类4	免疫耐受诱导及抑制剂形成机制			

**注** AAV：腺相关病毒；FⅨ：凝血因子Ⅸ；FⅧ：凝血因子Ⅷ。表中内容为DeepSeek根据关键词、聚类归属及关键词出现次数生成的概括结果，尚未经领域专家修订

对美国1 019篇相关文献进行关键词共现分析，共形成3个聚类（图2B）。DeepSeek将其分别概括为“血友病B型基因递送系统的优化与转化研究”“FⅧ抑制物清除策略及细胞治疗新路径”“基因治疗临床应用安全监测与卫生经济学评价”。DeepSeek进一步将美国文献数据集的特点概括为：“血友病B型研究领先，AAV介导的FⅨ基因治疗的动物模型与临床试验数据积累更多、免疫调控更系统化、真实世界数据先行，含united-states、health-care等关键词，凸显卫生政策与医保支付研究、基因编辑技术缺位，CRISPR相关词未入榜，以基因添加为主流”。针对该解读，我们分析CRISPR相关词未体现，可能是因为连接强度在整体研究体量中偏弱。我们进一步对CRISPR/Cas9应用于血友病治疗的相关文献进行了补充检索，共获得70篇文献，相关研究主要来自美国和中国（图3A）。

**图3:**
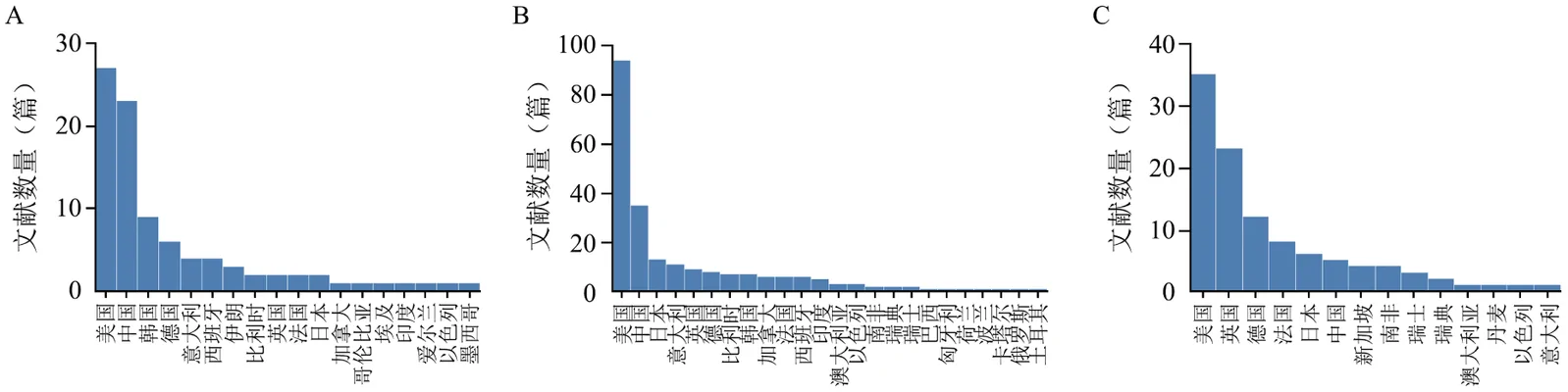
Web of Science核心数据库中血友病基因治疗特定研究方向的国家发文量分布 **A** CRISPR/Cas9相关研究；**B** 干细胞相关研究；**C** 宫内基因治疗相关研究

对中国174篇相关文献进行关键词共现分析，因整体研究体量较小，为了突显强关联的内容，将关键词最低出现次数设为5次，共形成3个聚类（表1）。DeepSeek将其分别概括为“AAV载体介导的FⅨ基因治疗体系构建与表达调控”“免疫屏障突破与细胞治疗新路径”和“FⅧ分泌体系重构及干细胞移植治疗”。中国特色体现在：“中国聚焦AAV介导的FⅨ基因治疗，美国同步深耕AAV载体递送FⅧ基因；中国在iPSCs、MSCs、HSCs的基因治疗研究热度远超美国”。为了验证干细胞在血友病基因治疗领域的研究现状，进一步在WOS中检索了相关研究，共获得167篇，其中美国文献占比超过50％，中国文献占比约20％（图3B）。

对英国172篇相关文献进行关键词共现分析，共形成2个聚类（表1）。DeepSeek将其概括为“AAV载体技术与胎儿期治疗探索”和“基因治疗临床转化与真实世界管理”，并将宫内基因治疗识别为英国文献数据集中较为突出的研究主题，提到“英国的研究特色是全球率先探索胎儿期治疗，在绵羊模型中验证子宫内基因治疗可行性”。为此，我们在WOS中检索血友病宫内基因治疗相关研究，发现全球相关研究68篇，其中美国35篇、英国23篇（图3C）。该结果提示，宫内基因治疗在英国相关研究中具有一定代表性，但该方向的美国文献数量仍多于英国。

三、CiteSpace全球血友病基因治疗研究热点及演进的DeepSeek解读

利用CiteSpace对全球血友病基因治疗相关文献进行关键词共现、聚类及突现分析。关键词聚类时间线图共展示7个主要聚类（#0～#6），聚类标签分别为“neutralizing antibodies”“recombinant factor viii”“hemophilia b”“coagulation factor ix”“factor viii”“hemophilia-a”和“endothelial-cells”（图4）。各聚类中的关键词沿时间轴排列，CiteSpace自动提取的聚类标签多为单个词或词组，仅能对聚类主题提供初步提示。DeepSeek将血友病基因治疗研究热点的演进过程概括为4个阶段，分别反映了不同时期的科研焦点与突破方向。

**图4:**
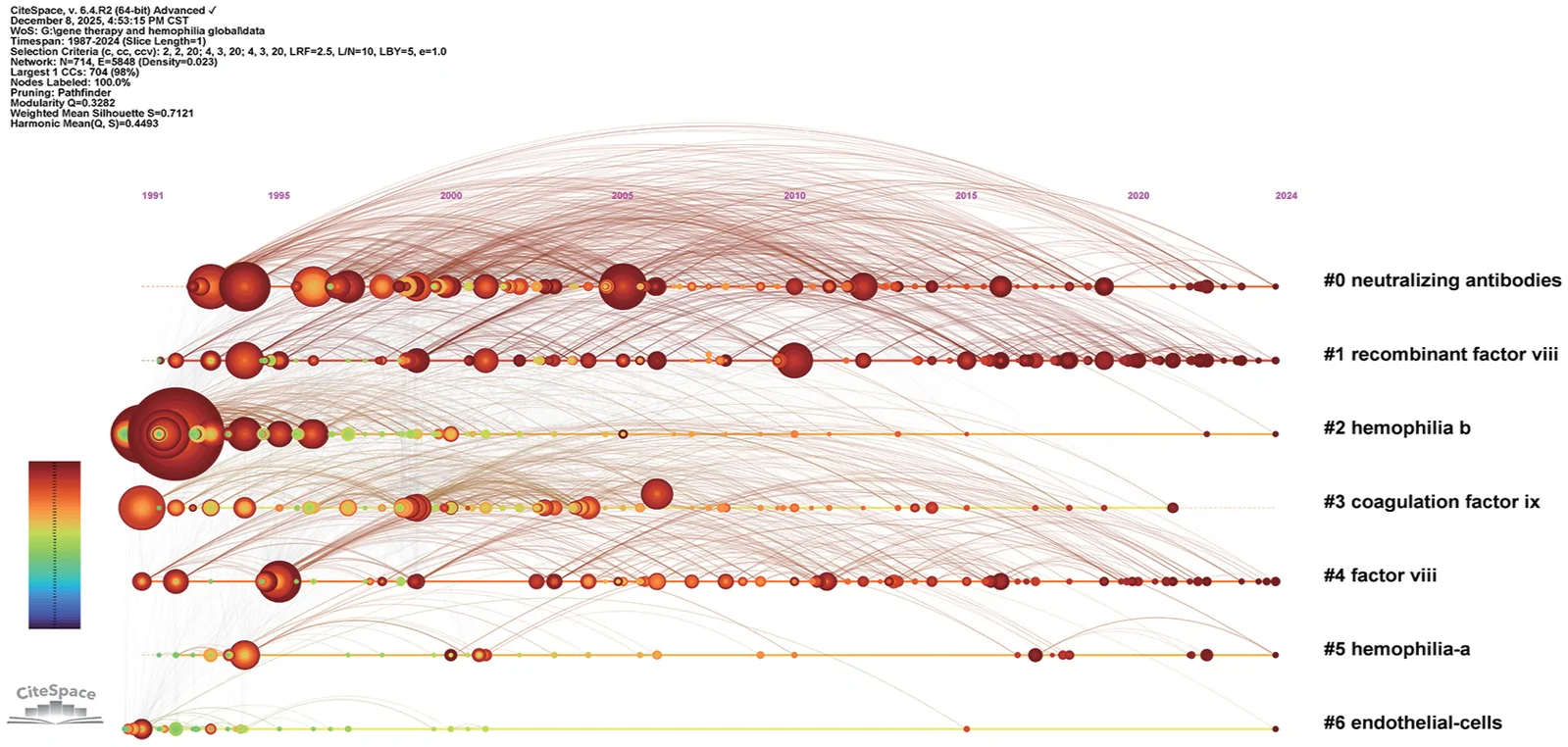
全球血友病基因治疗研究关键词聚类时间线图 **注** 基于CiteSpace关键词共现分析生成，共展示#0～#6共7个主要聚类，右侧聚类标签由CiteSpace自动提取，用于提示各聚类的主要内容

1. 技术探索期（1991–2000年）：突现关键词主要涉及human factor ix、transplantation、skin fibroblasts、hepatocytes、retroviral vector、mice和dogs等。DeepSeek据此将该阶段概括为载体开发及临床前模型验证，研究主要围绕逆转录病毒和AAV等递送工具，以及皮肤成纤维细胞、肝细胞和动物模型中的基因表达与表型验证展开。

2. 临床转化期（2001–2010年）：研究方向转向疗效优化与安全性评估。该阶段以AAV载体为主导，重点探索肝靶向递送与长期表型矫正，关键词如coagulation factor ix和immune response的突现强度显著，反映出因子表达持久性和免疫原性控制成为核心科学问题。

3. 临床验证期（2011–2020年）：研究重点进一步聚焦于安全性与疗效的临床化验证。大型临床试验的推进及免疫调控策略，如免疫耐受诱导技术的发展成为关键特征，高频关键词如efficacy、safety和clinical trial体现了该阶段对风险效益比的系统评估与规范化要求。

4. 精准化与患者获益期（2021–2024年）：研究热点转向个体化治疗与真实世界应用。以患者为中心的评价指标，如quality of life和patient相关关键词突现强度上升，反映出基因治疗与新型药物（如艾美珠单抗）的联合应用及健康技术评估等现实议题受到重视。

总体而言，DeepSeek基于CiteSpace导出的关键词时间与突现信息，将血友病基因治疗研究概括为由早期载体及动物模型探索，逐步向递送优化、临床验证和患者获益评价发展的演进过程。该阶段划分及具体解读的专业准确性由领域专家进一步评价。

四、领域专家对DeepSeek全球研究热点及演进趋势解读的评价

1. VOSviewer关键词聚类结果解读对比：DeepSeek与2位领域专家对全球VOSviewer关键词聚类结果解读比较见表2。DeepSeek和领域专家在4个聚类核心研究主题的判断上总体一致，分别涉及：病毒及非病毒载体开发、基因体内表达与动物模型验证；AAV载体递送优化以及预存中和抗体、免疫反应等问题；基因治疗临床试验中的疗效与长期安全性评价；FⅧ抑制物形成及免疫耐受诱导策略。该结果表明，DeepSeek能够依据聚类关键词及其出现次数识别和概括主要研究方向，其形成的总体主题框架与领域专家判断基本相符。DeepSeek与领域专家解读的差异主要体现在部分专业术语的使用上。例如，血友病领域通常使用“FⅧ抑制物”，而DeepSeek使用了“FⅧ抑制剂”这一表述。因此，DeepSeek的解读结果仍需由领域专家进行审核和规范，以保证专业术语及相关表述符合实际应用场景和行业规范。

**表2:** VOSviewer关键词共现结果DeepSeek与专家解读研究热点比较

聚类编号	Deepseek解读	专家1解读	专家2解读
聚类 1	**研究热点命名：**基因治疗载体的设计与体内表达调控。 **关键要点说明：**聚焦非病毒/病毒载体（腺病毒、慢病毒）的构建与优化，通过启动子设计、密码子优化等技术提升基因在体内的长效表达效率；结合新型递送系统解决靶向性问题；探索CRISPR等基因编辑工具的应用潜力。	**研究热点命名：**血友病A/B基础机制与基因递送系统研究。 **关键要点说明：**研究覆盖血友病A与B；形成从基因表达、体内验证到动物模型的完整基础研究链条；以AAV为核心的基因递送系统优化是研究重点。	**研究热点命名：**血友病基因治疗。 **关键要点说明：**研究焦点集中于血友病B和血友病A的基因治疗，探讨借助腺相关病毒等作为载体，在小鼠模型等中探讨基因导入后的表达效率、表型纠正以及可能引起的免疫反应。
聚类 2	**研究热点命名：**AAV载体递送策略及免疫原性突破。 **关键要点说明：**AAV载体主导研究，重点优化血清型的肝靶向递送效率；解决预存免疫（中和抗体）、免疫应答抑制及肝毒性问题；探索大动物模型中的长期疗效验证。	**研究热点命名：**AAV载体技术与凝血因子靶向表达研究。 **关键要点说明：**AAV载体技术是主流递送方案；肝脏与骨骼肌作为基因表达的核心靶器官到肝脏为主要靶器官。对免疫原性这一关键风险的研究重视。	**研究热点命名：**血友病基因治疗。 **关键要点说明：**以肝脏为靶器官逐渐代替以肌肉为靶标；腺相关病毒是主要载体；针对载体的中和抗体、转导效率和免疫反应是主要关注点。
聚类 3	**研究热点命名：**基因治疗临床转化与真实世界应用。 **关键要点说明：**关注Ⅲ期临床试验数据，评估长期安全性和有效性；探索与传统药物的联合策略；聚焦患者生活质量、医疗成本效益分析。	**研究热点命名：**基因治疗临床疗效与长期安全性评估。 **关键要点说明：**聚焦临床转化关键问题，研究关注临床疗效，尤其关注长期安全性。	**研究热点命名：**血友病基因治疗的临床研究。 **关键要点说明：**聚焦于血友病基因治疗的临床试验。
聚类 4	**研究热点命名：**免疫耐受诱导及抑制剂形成。 **关键要点说明：**研究抑制剂产生的免疫机制；开发免疫耐受策略；探索非病毒载体降低免疫原性的潜力；结合基因编辑敲除免疫原性表位。	**研究热点命名：**免疫耐受诱导与抑制物防治研究。 **关键要点说明：**聚焦血友病治疗过程中的抑制物产生难题；深入探讨T细胞与树突状细胞介导的免疫耐受机制，并提出诱导免疫耐受以恢复凝血功能的研究目标。	**研究热点命名：**FⅧ抑制物的处理。 **关键要点说明：**FⅧ抑制物阳性是血友病A治疗的难题；该聚类聚焦于通过诱导免疫耐受的策略来尝试解决这一难题。

**注** AAV：腺相关病毒；FⅧ：凝血因子Ⅷ

2. CiteSpace分析结果解读对比：DeepSeek与2位领域专家对CiteSpace分析结果的解读比较见表3，三方对血友病基因治疗领域总体发展脉络的判断基本一致。其一致性主要体现在以下3个方面：①载体演进主线：三方均认为AAV由早期探索阶段的载体之一，逐渐发展为该领域的主要递送工具，其技术优化推动了血友病基因治疗的临床转化。②免疫挑战的核心地位：DeepSeek与专家1均将免疫反应和中和抗体识别为临床转化阶段的重要障碍和持续关注点；专家2虽然未直接将其概括为免疫障碍，但提出采用预存中和抗体发生率相对较低的AAV5、AAV8等血清型进行策略优化，也体现了对该问题的关注。③临床试验：三方均关注临床试验特别是Ⅲ期临床试验的大规模开展，并认为安全性和有效性是评价血友病基因治疗临床应用的重要指标。

**表3:** CiteSpace关键词共现结果DeepSeek与专家解读研究热点演进趋势比较

对比维度	DeepSeek解读 （4个阶段）	专家1解读 （3个阶段）	专家2解读 （3个阶段）
阶段1			
时间与命名	技术探索期（1991–2000）	基础探索与动物验证/初步临床试验（1991–2001）	早期探索（1991–2001）
核心动向	载体开发与体外/动物模型验证	以小鼠/猪/狗模型为核心开展基因治疗的初步验证	以成纤维细胞、逆转录病毒、腺相关病毒等为载体，开展临床研究
关键进展	1. 逆转录病毒载体、AAV成为主流工具； 2. 靶细胞：皮肤成纤维细胞、肝细胞； 3. 动物模型：犬模型、小鼠模型成熟	1. 研究集中于FⅨ/FⅧ； 2. 通过皮肤成纤维细胞、肝细胞、肌肉细胞等靶细胞，以逆转录病毒、AAV为载体进行初步临床试验探索	1. 中国学者：1993年国际上率先报道将携带FⅨ的成纤维细胞埋入患者皮下，获得约1年表达，表达水平不太高。 2. 国外学者：以AAV2为载体肌注，获得表达但水平仍不理想
阶段2			
时间与命名	临床转化期（2001–2010）	载体技术优化与长期表达（2002–2010）	治疗策略优化（2002–2020）
核心动向	疗效优化与安全性挑战	以AAV为核心载体，重点解决基因表达持续性与安全性问题	以肝脏为靶器官，借助预存中和抗体发生率较低的AAV5、AAV8等进行策略优化
关键进展	1. AAV载体成为首选，肌内注射和肝靶向递送是关键途径； 2. 实现长期表型矫正和长期表达； 3. 免疫原性挑战显现：中和抗体、抑制剂成为焦点	国际上首个AAV载体血友病B基因治疗成功，并实现在一定时间内凝血因子的持续表达	国际上首次报道基因治疗可使血友病患者出血表型完全被纠正（至少一定时间内）
阶段3			
时间与命名	临床验证期（2011–2020）	临床转化加速与挑战应对期（2011–2024）	临床治愈（2021–2024）
核心动向	安全性与疗效临床化	从动物实验全面转向临床研究，中和抗体/预存抗体等问题受到关注	多个产品的临床试验获得成功，欧美药监部门批准相关产品上市
关键进展	1. 大型临床试验密集推进； 2. 免疫调控突破：免疫耐受诱导、FC融合蛋白； 3. 长期安全性成为核心评估指标	1. 继血友病B后，首个血友病A基因治疗获得成功； 2. 多个血友病A/B的AAV基因治疗产品获批国外上市	1. 国际上3家药企产品获FDA/EMA批准上市； 2. 血友病B：单次给药可完全纠正出血表型，FⅨ持续高水平表达； 3. 血友病A：相当部分受试者可较长时间实现FⅧ高水平表达
阶段4			
时间与命名	精准化与患者获益期（2021–2024）	–	–
核心动向	个体化治疗与真实世界应用	–	–
关键进展	1. 药物-基因联合策略（艾美珠单抗与基因疗法并行）； 2. 患者为中心研究（生活质量、护理、健康）； 3. 基因编辑技术进入视野	–	–
发展趋势总结	领域正从技术驱动转向患者价值驱动，未来需平衡长期安全性、可及性与创新技术（如基因编辑）	未明确给出总结性趋势判断	研究热点从早期探索演进到治疗策略优化，最终临床治愈成为可能（至少对部分患者而言）

**注** AAV：腺相关病毒；FⅧ：凝血因子Ⅷ；FⅨ：凝血因子Ⅸ；FDA：美国食品药品管理局；EMA：欧洲药品管理局。–：无该阶段

DeepSeek与领域专家解读的差异主要体现在阶段划分的细化程度，以及对领域历史背景和里程碑事件的呈现上。DeepSeek将2021–2024年单独划分为第四阶段，强调研究关注点向临床应用和患者获益拓展；两位专家则采用3阶段框架，将这一时期纳入第三阶段的延续，更侧重呈现由早期探索、治疗策略优化到临床应用的发展过程。DeepSeek依据emicizumab prophylaxis、care、cost等近期关键词，概括出研究内容逐渐向患者照护和治疗费用等方面扩展。领域专家则在文献计量数据的基础上补充了学科发展背景和历史事件，如中国学者1993年开展的成纤维细胞相关研究及相关里程碑进展，这些信息难以仅依据关键词数据获得。

五、美国、中国、英国研究热点的解读验证

将DeepSeek对美国、中国和英国VOSviewer关键词聚类结果的解读发送给领域专家审阅。专家认为，DeepSeek识别出的研究主题总体涵盖载体技术开发、免疫反应、临床试验的安全性与有效性评价以及抑制物处理等核心方向，与全球血友病基因治疗研究的主要热点基本一致。但在“细胞治疗”“干细胞治疗”和“CRISPR/Cas9”等新兴技术及治疗策略的解读中，存在概念混淆和过度概括现象。

在美国文献的解读中，DeepSeek将一个聚类概括为“FⅧ抑制物清除策略及细胞治疗新路径”。专家指出，造血干细胞治疗是一种新的治疗策略，与抑制物清除策略并不存在直接对应关系，将二者合并为同一研究主题属于概念混淆。对于DeepSeek提出的“基因编辑技术缺位，CRISPR相关词未入榜，以基因添加为主流”，专家认可基因添加仍为当前主要技术路径，但指出CRISPR/Cas9在血友病领域尚未广泛开展，可能与其现阶段的适用性有限有关。因此，CRISPR相关关键词未进入共现网络，并不能直接等同于美国在该方向不存在研究布局。

在中国文献的解读中，DeepSeek提出“中国聚焦AAV-FⅨ疗法，美国同步深耕AAV-FⅧ疗法”。专家指出，中国AAV-FⅧ疗法研究起步相对较晚，因而在现有文献中体现较少，但不能据此忽略中国在该方向的研究布局。对于“中国在iPSCs、MSCs和HSCs相关基因治疗方面的研究热度远超美国”的判断，专家认为相关主题在中国文献数据集中确实较为突出，但这种国家内部关键词的相对突出程度不能直接解释为中国在该方向领先美国。

总体来看，DeepSeek能够识别各国文献中的主要研究热点，但在解读新兴技术和提炼国家研究特色时，因缺乏充分的领域历史背景和外部参照，容易出现主题过度概括和跨国家不当比较。不同国家文献集的规模、关键词纳入数量及网络结构存在差异，各国数据集内部的关键词频次或聚类强度不具备直接横向比较的基础。通过在统一检索口径下对CRISPR/Cas9、干细胞和宫内基因治疗等方向进行补充检索，可以对DeepSeek提出的国家特色进行交叉验证。

## 讨论

VOSviewer和CiteSpace是广泛应用的文献计量分析工具，能够通过关键词共现、聚类及突现分析呈现特定领域的知识结构和研究趋势，但其输出结果主要以网络图谱、聚类标签及相关指标的形式呈现，对研究主题及演进脉络的深入解释仍需结合领域知识，通常需要领域专家投入较多时间进行归纳和判断。随着大语言模型能力的不断提升，利用其辅助解读文献计量分析结果，有望提高信息提取和知识归纳的效率，实现自动化或半自动化的研究热点挖掘，更好地服务于科研实践。本研究利用DeepSeek解读VOSviewer和CiteSpace生成的关键词共现、聚类及突现分析数据，概括血友病基因治疗领域的研究热点并梳理其演进脉络。同时，邀请领域专家基于相同数据进行独立解读，通过比较DeepSeek与专家解读的一致性和差异，评价DeepSeek辅助解读文献计量数据的可行性及其应用边界。

可行性方面，在研究热点识别上，DeepSeek表现出较好的信息提取和概括能力，其对研究主体框架的解读与领域专家的判断基本一致。与人工解读相比，DeepSeek能够较快地处理结构化的关键词聚类数据，生成层次较为清晰、概括性较强的解读结果，有助于科研人员快速把握不同聚类之间的主题联系，形成对领域知识结构的整体认识。在研究热点演进趋势的解读上，DeepSeek能够依据关键词首次出现年份、突现强度、突现起止时间及聚类归属等信息，概括出血友病基因治疗由早期技术探索、载体与治疗策略优化逐步走向临床验证和患者获益评价的发展主线。其对AAV载体演进、免疫反应和中和抗体等关键问题，以及临床试验中安全性和有效性评价的认识，与领域专家的解读基本一致。此外，DeepSeek能够同时处理较多关键词及其时间信息，并关注emicizumab prophylaxis、quality of life、care和cost等近期出现或突现的关键词，据此提示研究关注点可能正在由载体和疗效等技术问题，逐渐向患者生活质量、临床照护和治疗费用等方面拓展。在专家提出的3阶段框架基础上，DeepSeek进一步将2021–2024年概括为“临床应用与患者获益关注期”，为理解近期研究热点变化提供了补充视角。但这一阶段划分属于模型基于关键词数据作出的归纳，其合理性仍需结合领域知识和后续研究加以判断。

局限性方面，DeepSeek为形成概括性结论，可能弱化具体的专业语境，在专业术语的规范性方面仍存在不足。例如，将血友病领域通常使用的“FⅧ抑制物”表述为“FⅧ抑制剂”，将“艾美赛珠单抗”表述为“艾美珠”。因此，模型输出仍需由领域专家进行术语审核和规范。此外，DeepSeek主要依据关键词频次、突现强度和聚类归属等结构化数据进行判断，缺乏充分的领域知识和历史背景，对历史事件重要性的判断容易受到数据表现强弱的影响。在领域价值判断及里程碑事件识别方面，模型难以像专家一样结合学科发展背景，赋予“首个中国报道”以应有的历史地位。对于国家这种局部数据，尽管可以识别核心热点问题，但也存在新兴方向上的过度解读和强行国家间比较造成的国家特色误判的风险。所以，通过引入独立的、基准化的宏观文献数据（如全球发文统计、国家份额），才能有效纠正这些偏差，这是确保后续分析逻辑严谨性的关键步骤。这一验证也表明，跨国比较分析不能依赖于大语言模型对单一国家聚类结果的直接解读，而必须依赖人工引入外部数据和领域知识进行校准与检验，比如本研究提到的干细胞、CRISPR/Cas9与宫内基因治疗。

综上所述，本研究初步探讨了大语言模型辅助解读文献计量分析结果的可行性。在全球文献数据分析中，DeepSeek对领域研究热点及发展脉络的总体判断与领域专家基本一致，表明其可作为文献计量结果解读的辅助工具。大语言模型能够快速处理结构化数据并形成较为系统的分析框架，领域专家则能够结合专业语境、学科历史和里程碑事件，对模型输出进行校准和补充。二者相互协作，有助于提高文献计量分析结果的解读效率和专业性。

在单一国家文献数据的分析中，DeepSeek可能依据各国数据集内部的关键词频次或聚类强度，作出某一研究方向“领先”或“缺失”的判断，进而导致国家研究特色的误判。因此，应用大语言模型开展跨国研究热点比较时，应引入统一检索口径下的全球文献数据，并结合领域专家意见进行校准。随着大语言模型的持续迭代，后续研究可进一步比较不同主流模型对文献计量数据的解读表现，并探索通过补充领域背景、全球参照数据及优化提示词等方式，降低模型在新兴研究方向解读和跨国比较中的过度概括与以偏概全风险。

## Supplementary Material


